# An initial comparative study on the antennal morphology of Zoraptera (Insecta) with special reference to the sensilla

**DOI:** 10.1038/s41598-025-08212-x

**Published:** 2025-07-05

**Authors:** Michel J. Faucheux, Gabriela Pačková, Petr Kočárek, Robin Kundrata

**Affiliations:** 1 Laboratoire d’Endocrinologie des Insectes Sociaux, Faculté des Sciences et des Techniques, Université de Nantes, 2 Rue de la Houssinière, B.P. 92208, 44322 Nantes Cedex 03, France; 2 Department of Zoology, Faculty of Science, Palacky University, 17. Listopadu 50, 77146 Olomouc, Czech Republic; 3 Department of Biology and Ecology, Faculty of Science, University of Ostrava, Chitussiho 10, 71000 Ostrava 2, Czech Republic

**Keywords:** Scanning electron microscopy, Polyneoptera, Sensilla, *Spermozoros*, *Spiralizoros*, Zoology, Entomology

## Abstract

Zoraptera represent one of the smallest and least-known insect orders. They live mainly in tropical and subtropical forests and have a cryptic lifestyle. To obtain a better understanding of the detailed antennal morphology and its potential use for taxonomic research in this group, for the first time we conducted a scanning electron microscopy study of the antennal sensilla of Zoraptera. We examined two species of *Spiralizoros* (Spiralizoridae) and a single species of *Spermozoros* (Zorotypidae). We identified 10 different sensilla structures belonging to five main types in Zoraptera. While Böhm sensilla, sensilla campaniformia, sensilla chaetica (subtypes C1–C2), sensilla trichodea, and sensilla basiconica (subtypes B1–B2) were present in all species, sensilla chaetica C3, sensilla basiconica B3 and sensilla styloconica were present only in *Spermozoros*. We discussed the possible functions of all observed sensilla based on their external morphology. Additionally, we preliminarily compared the variability of antennal sensilla between the families as well as two species of the same genus, and investigated the differences between both sexes as well as apterons and dealates of the same species. This study provides the first step toward future research on antennal morphology within Zoraptera and its significance for their systematics.

**Supplementary Information:**

The online version contains supplementary material available at 10.1038/s41598-025-08212-x.

## Introduction

Zoraptera (angel insects, groundlice) are minute, inconspicuous polyneopteran insects occurring mainly in tropical regions although some are known from subtropical or temperate areas^1,2^. They have a cryptic lifestyle and are usually found in rotting wood, under bark or exceptionally under stones^1,3^. Zorapterans are subsocial, having two forms; wingless, eyeless and weakly pigmented (apterons sensu Engel^4^), or winged, with ocelli and compound eyes and a distinct pigmentation (alates). Representatives of the latter form shed wings over time (dealates). Both nymphs and adults feed on fungal hyphae and spores or are scavengers^1,5^.

Although they represent an old evolutionary lineage with a Paleozoic origin^6–9^, Zoraptera belong to the least numerous insect orders. They comprise only 47 extant and 15 fossil species^2,10^. However, based on the recent research and estimates the diversity of Zoraptera is likely much greater than what is currently known^1,3,5,7,11–13^. The phylogenetic position of this order is a matter of debate (i.e., the so-called “Zoraptera problem” introduced by Beutel and Weide^14^); recently, phylogenomic studies found that Zoraptera are either sister to Dermaptera^6,8,15–17^ or all remaining Polyneoptera^9^. The extreme uniformity in the external morphology of Zoraptera representatives led to conservative classification with a single nominotypical genus, *Zorotypus* Silvestri, 1913, in a single family, Zorotypidae^18^. This classification was then widely used for more than a century. In 2020, two research teams independently used a combination of nuclear and mitochondrial markers to reveal the phylogenetic relationships within Zoraptera^7,11^. Both studies identified two main lineages, which Kočárek et al.^11^ named Zorotypidae and Spiralizoridae, each of which has two subfamilies. This classification was further supported by the morphological synapomorphies, especially male genitalia, the apex of the male abdomen, and metatibial spurs^11^. Recently, fossil species have been classified into the currently used system based on the available morphological evidence^10^.

Considering the generally uniform external morphology of Zoraptera from different phylogenetic lineages, more detailed morphological characters should be investigated for their potential in diagnosing zorapteran groups at various taxonomic levels. The sensillar equipment on antennae may provide important diagnostic characters that can be used for reliable species identification as well as for the construction of a phylogenetic hypothesis for a given group, as demonstrated for some other insect lineages^19–23^. No comparative study on the morphology of antennae has ever been performed for Zoraptera. To date, the only work focused on the antennal sensilla of this order is that by Slifer and Sekhon from 1978^24^. They studied the sensory receptors on the antennal flagellum of *Zorotypus hubbardi* Caudell, 1918 (currently in *Usazoros*) from Florida, USA, and compared them with those of termites.

**Fig. 1:**
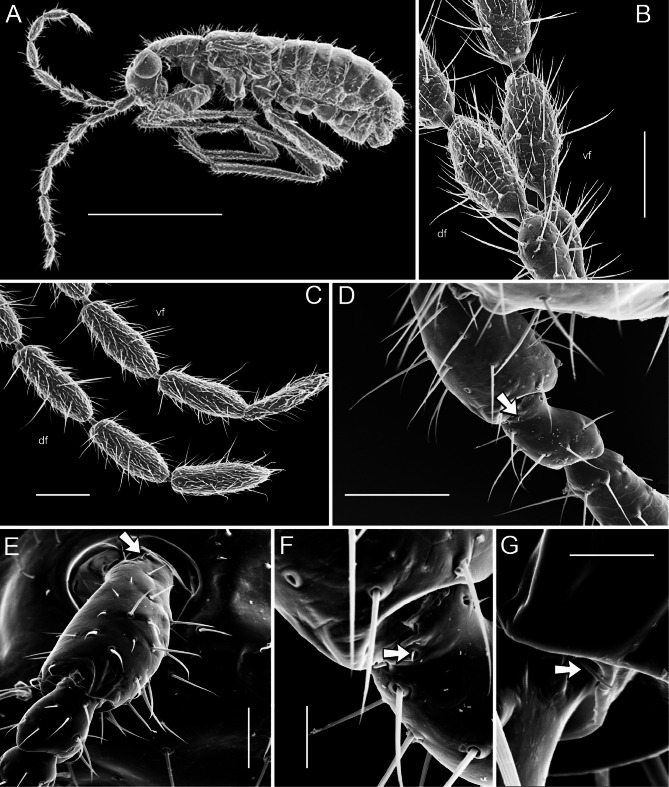
Scanning electron microscopy images of antennal morphology of Zoraptera. (**A**) *Spiralizoros* sp., female dealate, whole body. (**B**) *Spiralizoros* sp., female apteron, antennomeres 3–5. (**C**) *Spermozoros weiweii*, female apteron, antennomeres 6–9. (**D**) *Spiralizoros cervicornis*, male dealate, antennomeres 1–3, arrow points to Böhm sensillum on pedicel. (**E**) *Spiralizoros cervicornis*, male apteron, antennomeres 1–3, arrow points to Böhm sensillum on scape. (**F**) *Spiralizoros cervicornis*, male dealate, scape and pedicel, arrow points to Böhm sensillum on pedicel. (**G**) *Spiralizoros cervicornis*, male apteron, scape and pedicel, detail of Böhm sensillum (arrow) near sensillum chaeticum. Scale bars: (**A**) 1 mm, (**B**–**D**) 100 µm, (**E**) 50 µm, (**F**) 20 µm, (**G**) 10 µm. Abbreviations: df, dorsal face; vf, ventral face.

**Fig. 2:**
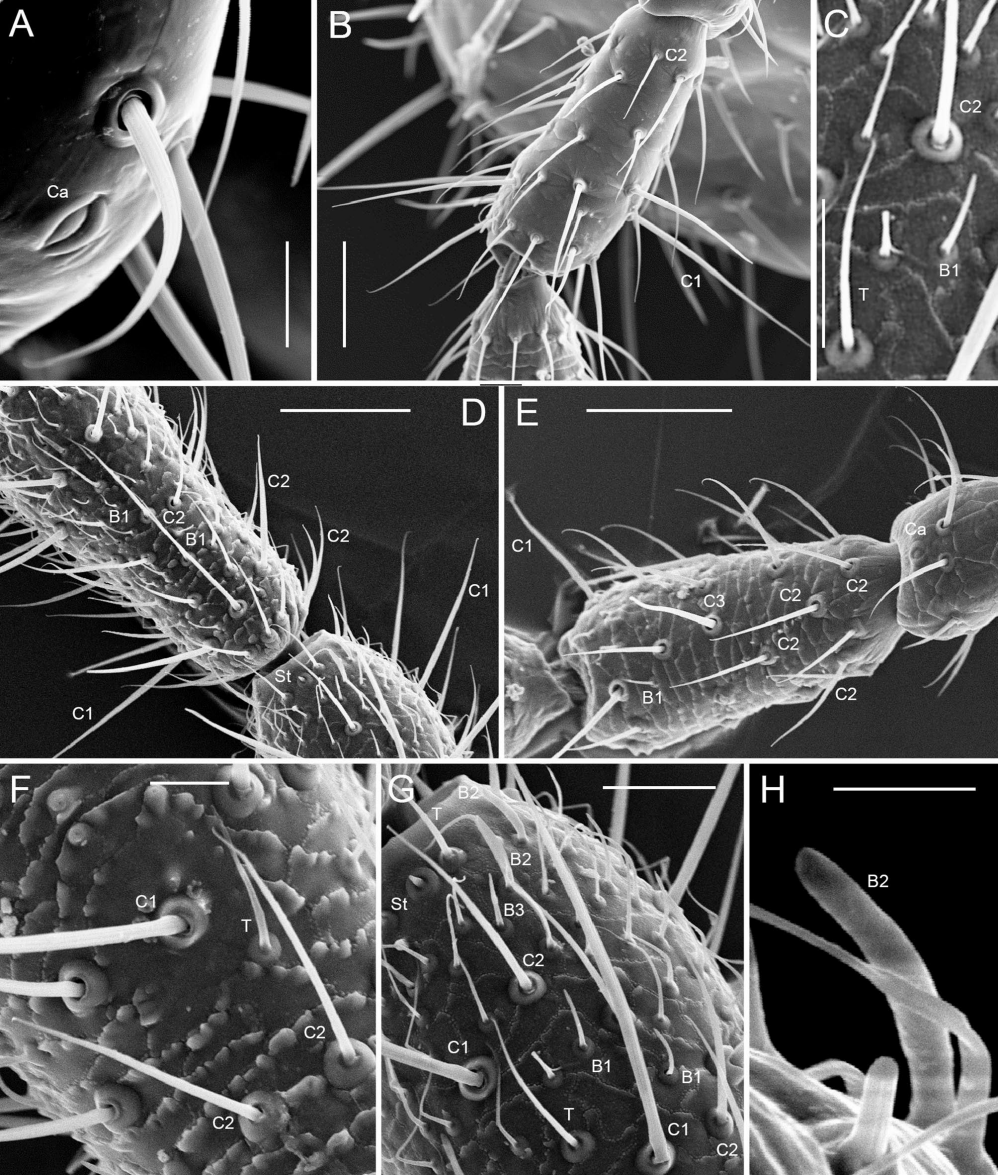
Scanning electron microscopy images of antennal morphology of Zoraptera. (**A**) *Spiralizoros cervicornis*, male apteron, pedicel, detail of sensillum campaniformium. (**B**) *Spiralizoros cervicornis*, male apteron, antennomeres 2–4. (**C**) *Spermozoros weiweii*, male apteron, antennomere 7, details of various sensilla. (**D**) *Spermozoros weiweii*, male apteron, antennomeres 7–8. (**E**) *Spermozoros weiweii*, male apteron, antennomeres 2–4. (**F**) *Spermozoros weiweii*, male apteron, antennomere 7, surface and sensilla chaetica C1 and C2. (**G**) *Spermozoros weiweii*, male apteron, antennomere 7, various sensilla. (**H**) *Spiralizoros cervicornis*, male apteron, antennomere 9, detail of sensillum basiconicum 2 and broken sensillum chaeticum 2. Abbreviations: B1–B3, sensilla basiconica 1–3; C1–C3, sensilla chaetica 1–3; Ca, sensillum campaniformium; St = sensillum styloconicum; T, sensilla trichodea. Scale bars: (**A**, **F**) 10 µm, (**B**, **D**, **E**) 50 µm, (**C**, **G**) 20 µm, (**H**) 5 µm.

In this initial study devoted to the comparative antennal micromorphology of Zoraptera, we used scanning electron microscopy to examine male and female apterons and dealates of Bornean species from two genera, *Spiralizoros* Kočárek, Horká and Kundrata, 2020 and *Spermozoros* Kočárek, Horká and Kundrata, 2020 (Figs. 1, 2; Table S1), which represent both currently recognized families (Spiralizoridae and Zorotypidae, respectively)^11^. Our main goals were to identify the types of antennal sensilla in Zoraptera; discuss their possible functions; preliminarily compare the variability in morphology, number, and distribution of antennal sensilla between the families; compare two species of the same genus (*Spiralizoros*); and investigate the differences between the apterons and dealates of the same species. We also compared our results with those of Slifer and Sekhon^24^.

## Results and discussion

### Gross morphology of antennae in Zoraptera

The length of the antennae of the three examined species varied from 1.3 to 1.7 mm (Table S2). For all the species, the antennae were shorter than the body (Fig. 1A), and those of the males were shorter than those of the females, which corresponded with their body lengths. The antennae of Zoraptera have nine antennomeres and consist of a large scape (antennomere A1), a small pedicel (antennomere A2), and a flagellum composed of seven flagellomeres (antennomeres A3–A9), which are longer than wide (Fig. 1A–E). In all cases, the pedicel (Fig. 1D,E) was the smallest antennomere, usually followed by antennomere 3 (with the exception of the male apteron of *Spiralizoros* sp. where the scape was shorter than antennomere 3). Antennomere 5 was the longest in all the specimens except for the female apterons of both *Spiralizoros* species (Table S2).

The flagellomeres are ovoid in shape and there are distinct constrictions or necks between them (Fig. 1B,C). These narrow necks must not only allow the passage of nerves, blood vessels and trachea but also allow a certain degree of mobility of flagellomeres against each other. The short distal part of the neck, which is fixed on the deformable joint membrane, is differently oriented, whereas the proximal long part of the following flagellomere remains rigid. The dimensions of the constrictions are variable; they are larger in the proximal flagellomeres than in the distal flagellomeres. For example, in the female apteron of *Spermozoros weiweii* (Wang, Li and Cai, 2016)^25^, the length of neck, proximal diameter, and distal diameter (in µm) for A4–A5 and A7–A8 are 20.5 : 16.8 : 23.8 and 18.2 : 10.0 : 13.6, respectively (Fig. 1C). The first segment of the flagellum (A3) of all examined specimens was shorter than all the remaining flagellomeres (Table S2).

The antennal cuticle on the scape and the pedicel was smooth. Some cuticular scales may rarely appear on the ventral face of the first flagellomere, such as in the male apteron of *Spiralizoros cervicornis*^26^ (Fig. 2B). The cuticular scales occupy the whole surface of flagellomeres 2–7 (Fig. 2D,E).

### Sensilla on scape and pedicel (A1–A2)

The scape and the pedicel bear Böhm sensilla and sensilla campaniformia, which are not found further on flagellomeres, and sensilla chaetica of various lengths and shapes. Böhm sensilla and sensilla campaniformia were not reported for *Usazoros hubbardi* by Slifer and Sekhon because those authors did not study the scape and the pedicel but focused chiefly on the flagellomeres. In this study we focus mainly on the flagellomeres but below we also briefly mention our observations of the sensilla on the scape and pedicel.

#### Böhm sensilla (BS)

Böhm sensilla are short (usually 1.7–3.1 µm), smooth, thorn-like, aporous sensilla located in an off-center position in a socket (Fig. 1F,G). This type of sensilla is located at the base (condyle) of the scape and pedicel, as in other insects. While BS are usually found in three clusters on the scape and in two clusters on the pedicel among many insects^19,20^, in which the mean numbers vary between 25 and 100 sensilla^27^, the BS of zorapterans are much less conspicuous and occur in very low numbers. For example, in the male apteron of *Spiralizoros cervicornis*, a cluster of three BS is located ventrally at the base of the scape (Fig. 1E) and a single sensillum is visible at the base of the pedicel (Fig. 1G). Interestingly, the BS on the scape are longer and sharper than usual BS of other zorapterans; they measure 5.0 µm, 6.2 µm, and 15.3 µm, respectively, and project almost perpendicularly to the surface of the scape. The male dealate of *S. cervicornis* has at least four BS located on the pedicel (Fig. 1D). The pedicellar BS have the same morphology as those on the scape.

Böhm sensilla of insects function as proprioceptors that monitor antennal movements and positions via a reflex mechanism^19,27–29^. The low numbers of BS in Zoraptera suggest that their role may not be as important as that in some other insects.

#### Sensilla campaniformia (Ca)

In the examined zorapterans, these sensilla are located only on the inner edges of the scape and pedicel, respectively, and are present in low numbers. For example, in the male apteron of *Spiralizoros cervicornis*, there are three sensilla campaniformia: the first in the proximal third of scape, the second at the distal edge of the scape, and the third at the distal edge of the pedicel (Figs. 1E; 2A,E). All three sensilla are identical; with a central oval dome embedded in the integument, surrounded by an oval ring of raised cuticle which is divided into two parts (Fig. 2A). In the center of the dome, there is an apparent closing molting channel which is characteristic for the hemimetabolous insects^30^. The dimensions of the dome (large diameter × small diameter) for the above-mentioned three sensilla Ca are as follows: 3.6 × 1.7 µm, 7.6 × 3.1 µm, and 4.8 × 1.9 µm, respectively. The dimensions of the ring (large diameter × small diameter) are 7.1 × 4.8 µm, 10.7 × 6.0 µm, and 8.3 × 4.3 µm, respectively. These sensilla are all longitudinally oriented.

Sensilla campaniformia are mechanoreceptors with proprioceptive functions that respond to stress and strain in the exoskeletons of insects, and are mostly concentrated near the antennal joints^28,31^. The campaniform sensilla with round domes respond to strains in the cuticle from all directions whereas the sensilla with oval domes are directionally selective, i.e., respond to the forces that have a compression component of shear in the direction of their long diameter^32^. Since three sensilla Ca of zorapterans have the same orientation and are close to each other, they constitute a group that may act as a single unit^32^.

#### Sensilla chaetica (C)

Like in other insects, sensilla chaetica on the scape and pedicel vary widely in shape and length (Figs. 1D–F; 2A), and they become more or less stable only on the flagellum. Although some sensilla chaetica on the scape and pedicel of Zoraptera resemble flagellar sensilla C1 and C2 (see below), their lengths and distributions differ. For example, on the ventral face of the scape of the male apteron of *Spiralizoros cervicornis* (Fig. 1E), we observed two straight, perpendicularly erect sensilla near the base, being 17.3 and 24.1 µm long, respectively, at least a dozen of sensilla ranging from those more or less straight but with a curved tip (28.8–43.1 µm long) to those more or less curved (7.7–33.4 µm long), and a single thin sensillum (16.3 µm long), with a basal diameter equal to one third of that of the remaining sensilla chaetica on the scape.

### Sensilla on the antennal flagellum (A3–A9)

We identified at least five types or subtypes of sensilla on the antennal flagellum of all the studied species (including both sexes and forms): sensilla chaetica with two subtypes, 1 and 2 (C1 and C2), sensilla trichodea (T), and sensilla basiconica with two subtypes, 1 and 2 (B1 and B2). Furthermore, *Spermozoros weiweii* had three additional (sub)types, present in both sexes: sensilla chaetica subtype 3 (C3), sensilla basiconica subtype 3 (B3), and sensilla styloconica (St) (Figs. 1, 2; Tables 1, 2, 3, 4 and 5, S3–S10).

**Table 1:** Length (upper value) and basal width (lower value) of all sensilla (sub)types on the antennal flagellum for all examined species of Zoraptera (mean ± SE, in µm).

Species / Sensillum type	C1	C2	C3	T	B1	B2	B3	St
*Spiralizoros* sp. male apteron	68.9 ± 4.3	34.8 ± 2.9	–	28.7 ± 5.2	19.9 ± 2.1	18.2 ± 0.3	–	–
	2.1 ± 0.2	1.5 ± 0.2	–	0.7 ± 0.1	0.5 ± 0.1	2.5 ± 0.2	–	–
*Spiralizoros* sp. female apteron	72.7 ± 5.1	41.6 ± 1.8	–	32.4 ± 4.6	20.3 ± 1.9	18.1 ± 0.7	–	–
	2.0 ± 0.2	1.5 ± 0.3	–	0.7 ± 0.1	0.6 ± 0.1	2.5 ± 0.1	–	–
*Spiralizoros* sp. female dealate	70.1 ± 3.9	36.5 ± 2.7	–	34.9 ± 3.2	17.3 ± 1.5	18.2 ± 0.4	–	–
	2.1 ± 0.1	1.4 ± 0.2	–	0.7 ± 0.2	0.5 ± 0.1	2.5 ± 0.1	–	–
*S. cervicornis* male apteron	57.8 ± 5.9	42.3 ± 3.1	–	28.9 ± 2.4	13.6 ± 1.7	10.5 ± 1.3	–	–
	2.1 ± 0.1	1.6 ± 0.1	–	1.0 ± 0.1	0.7 ± 0.1	2.1 ± 0.2	–	–
*S. cervicornis* female apteron	125.0 ± 4.3	70.6 ± 2.3	–	58.1 ± 3.7	21.3 ± 1.5	20.0 ± 1.9	–	–
	1.8 ± 0.1	1.6 ± 0.1	–	0.9 ± 0.2	0.6 ± 0.1	2.5 ± 0.1	–	–
*S. cervicornis* male dealate	78.9 ± 5.2	29.4 ± 1.8	–	28.7 ± 1.9	17.9 ± 2.1	15.4 ± 1.8	–	–
	2.3 ± 0.2	1.5 ± 0.1	–	1.1 ± 0.1	0.6 ± 0.2	2.3 ± 0.2	–	–
*Spermozoros weiweii* male apteron	55.0 ± 5.1	32.7 ± 3.4	37.8 ± 5.2	31.1 ± 2.7	15.6 ± 1.9	14.3 ± 0.4	11.4 ± 0.1	3.5 ± 0.1
	2.9 ± 0.2	1.7 ± 0.1	3.2 ± 0.2	1.2 ± 0.2	0.6 ± 0.1	2.4 ± 0.1	1.2 ± 0.1	1.5 ± 0.1
*Spermozoros weiweii* female apteron	62.5 ± 4.8	35.2 ± 2.6	36.3 ± 4.1	32.3 ± 3.4	12.1 ± 2.3	14.5 ± 0.2	10.1 ± 0.2	3.6 ± 0.1
	2.8 ± 0.1	1.7 ± 0.1	3.0 ± 0.1	1.2 ± 0.1	0.6 ± 0.1	2.3 ± 0.1	1.2 ± 0.2	1.5 ± 0.1

**Table 2:** Numbers of sensilla on flagellomeres (A3–A9) of *Spiralizoros* sp.

Sensilla	Sex, form	A3	A4	A5	A6	A7	A8	A9	Total
Chaetica C1	M apt	13	11	14	17	16	12	14	136
	F apt	16	10	12	10	9	13	12	134
	F dea	11	11	11	12	13	14	10	138
Chaetica C2	M apt	31	15	30	38	26	34	30	204
	F apt	14	23	21	23	26	12	25	144
	F dea	23	48	45	36	38	31	32	253
Trichodea	M apt	12	40	52	57	68	55	51	335
	F apt	4	37	43	55	61	43	46	289
	F dea	1	37	52	68	80	74	57	369
Basiconica B1	M apt	–	16	11	20	15	11	9	82
	F apt	–	17	10	10	5	8	10	60
	F dea	–	11	8	9	8	10	7	53
Basiconica B2	M apt	–	–	–	3	7	2	3	15
	F apt	–	5	3	5	4	3	2	22
	F dea	2	3	4	2	1	3	2	17
Total		127	284	316	365	377	325	310	2251

M, male; F, female; apt, apteron; dea, dealate.

**Table 3:** Numbers of sensilla on flagellomeres (A3–A9) of *Spiralizoros cervicornis*. M = male, F = female, apt = apteron, dea = dealate.

Sensilla	Sex, form	A3	A4	A5	A6	A7	A8	A9	Total
Chaetica C1	M apt	13	14	17	20	16	18	19	168
	F apt	15	15	17	18	14	13	17	129
	M dea	20	16	22	23	15	16	12	157
Chaetica C2	M apt	11	22	29	21	19	22	14	138
	F apt	24	26	15	28	23	24	14	154
	M dea	7	14	16	16	23	17	12	106
Trichodea	M apt	6	33	42	44	36	40	31	232
	F apt	7	28	32	36	27	44	43	217
	M dea	–	30	23	23	25	20	35	156
Basiconica B1	M apt	2	38	67	73	52	52	81	365
	F apt	4	24	37	63	61	36	53	278
	M dea	1	51	75	69	59	60	47	362
Basiconica B2	M apt	1	2	2	4	5	6	17	37
	F apt	–	–	–	–	1	1	4	6
	M dea	3	23	14	11	14	10	11	86
Total		114	336	408	449	390	379	410	2588

**Table 4:** Numbers of sensilla on flagellomeres (A3–A9) of *Spermozoros weiweii*.

Sensilla	Sex, form	A3	A4	A5	A6	A7	A8	A9	Total
Chaetica C1	M apt	10	10	14	16	8	13	11	132
	F apt	15	12	13	10	11	13	14	128
Chaetica C2	M apt	20	32	17	28	20	22	10	149
	F apt	17	29	15	15	27	28	13	154
Chaetica C3	M apt	–	2	1	2	3	1	2	11
	F apt	1	1	1	1	1	1	1	7
Trichodea	M apt	5	24	118	105	63	52	46	413
	F apt	11	30	79	60	31	27	42	280
Basiconica B1	M apt	1	43	29	48	51	48	45	265
	F apt	8	22	26	46	84	85	44	315
Basiconica B2	M apt	–	6	7	7	15	10	5	50
	F apt	1	3	5	3	4	8	4	28
Basiconica B3	M apt	–	–	1	3	3	2	2	11
	F apt	–	–	–	3	2	2	2	9
Styloconica	M apt	1	1	1	1	–	1	3	8
	F apt	1	1	–	1	1	–	1	5
Total		91	216	327	349	324	313	245	1965

M, male; F, female; apt, apteron; dea, dealate.

**Table 5:** Percentages of various (sub)types of sensilla on flagellomeres of Zoraptera. C1–C3, sensilla chaetica of subtypes 1–3; T, sensilla trichodea; B1–B3, sensilla basiconica of subtypes 1–3; St, sensilla styloconica.

Species / Sensillum type	C1	C2	C3	T	B1	B2	B3	St
*Spiralizoros* sp. male apteron	17.6	26.4	–	43.4	10.6	2.0	–	–
*Spiralizoros* sp. female apteron	20.7	22.2	–	44.5	9.2	3.4	–	–
*Spiralizoros* sp. female dealate	16.6	30.5	–	44.5	6.4	2.0	–	–
*S. cervicornis* male apteron	17.9	14.7	–	24.7	38.8	3.9	–	–
*S. cervicornis* female apteron	16.4	19.7	–	27.7	35.4	0.8	–	–
*S. cervicornis* male dealate	18.1	12.2	–	17.4	42.4	9.9	–	–
*Spermozoros weiweii* male apteron	12.7	14.3	1.1	39.7	25.5	4.8	1.1	0.8
*Spermozoros weiweii* female apteron	13.8	16.6	0.8	30.2	34.0	3.0	1.0	0.6

#### Sensilla chaetica (C1–3)

These are long stiff hairs which are inserted in a large basal socket. Two basic subtypes (C1 and C2) are present in all the studied species, whereas the third subtype (C3) is present only in *Spermozoros*. Sensilla chaetica constitute 42.9–47.1% of all antennal sensilla in *Spiralizoros* sp., 30.3–36.1% in *Spiralizoros cervicornis*, and 28.1–31.2% in *Spermozoros weiweii* (Table 5).

Sensilla chaetica C1 are, due to their length, the most conspicuous sensilla in zorapteran antennae (Figs. 1B–D, 2B, D, E). Most of them encircle each flagellomere at two levels: at approximately the first quarter and after the first half. Each circle usually contains 6–7 more or less regularly spaced C1, which are oriented almost perpendicularly to the long axis of the antenna. These sensilla are usually 50–120 µm long and approximately 2 µm wide at the base and 0.7 µm wide at the apex (Table 1). They are in large sockets whose inner and outer diameters reach 3.8 µm and 6.7 µm, respectively, with a height of 3.2 µm. The hair itself is longitudinally striated, slightly curved, gradually tapered toward the tip, and quite frequently characteristically swollen at the level of the socket (Fig. 2F,G). There is no terminal pore visible. Based on our results, *Spermozoros weiweii* has shorter sensilla C1 than *Spiralizoros* spp., *Spiralizoros cervicornis* generally has longer sensilla C1 than *Spiralizoros* sp., and females of all species have longer sensilla C1 than their male counterparts (Table 1). In terms of the morphological forms, there were no significant differences in the length of sensilla C1 between the female apteron and the dealate in *Spiralizoros* sp., whereas in *S. cervicornis*, the male dealate had significantly longer sensilla C1 than the apteron of the same sex did (Table 1).

Due to the length of sensilla chaetica C1, antennae greatly expand their field of action. Sensilla C1 studied on *Usazoros hubbardi* were identified as "thick-walled chemoreceptors" by Slifer and Sekhon^24^ (Tables 6, S11). They stained the antenna externally with a 0.5% solution of crystal violet^33^ and wrote that the "stain enters through the tip [of thick-walled chemoreceptors] when dye is applied to the intact insect". Such sensilla would be contact chemoreceptors or gustatory receptors. Our findings, however, do not support this interpretation. No terminal pore was observed via SEM. Although the terminal pore of sensilla may often be difficult to observe, based on our experience, the elongated and narrowed distal part of sensilla, as in C1, usually does not allow the presence of a pore. In the antennae of insects studied to date, contact chemoreceptors are always relatively stocky sensilla with a blunt tip whose general shape is different from that of sensilla C1 of Zoraptera. In addition, sensilla with uniporous sensory cuticles have an external appearance of hairs that are usually short to medium-long, pegs, papillae, small plates, or, simply, pores in a cuticular depression^34^. We hypothesize that sensilla chaetica C1 of zorapterans may be tactile mechanoreceptors that play a role in communicating with the environment. A certain sensitivity to air currents (vibroreception) is also possible for these long sensilla. Future transmission electron microscopic studies of sensilla C1 are crucial for revealing the presence or absence of neuronal dendrites in hair. The absence would characterize the mechanoreceptors in which the dendrite terminates as a tubular body at the base of a hair^35^.

**Table 6:** Antennal sensillum (sub)types in Zoraptera. Information on antennae of *Usazoros hubbardi* was taken from Slifer and Sekhon.

Sensillum type/species	*Spiralizoros* spp.	*Spermozoros weiweii*	*Usazoros hubbardi*
*S. chaetica *C1	+	+	B, thick-walled chemoreceptors
*S. chaetica* C2	+	+	A, tactile hairs
*S. chaetica* C3	−	+	−
*S. trichodea*	+	+	E, long, slender thin-walled chemoreceptors
*S. basiconica* B1	+	+	D, short, slender thin-walled chemoreceptors
*S. basiconica* B2	+	+	C, wide thin-walled chemoreceptors
*S. basiconica* B3	−	+	−
*S. styloconica* St	−	+	−

Sensilla chaetica C2 are semierect at an angle of approximately 30 degrees and are oriented distad. These sensilla are shorter than sensilla C1 (usually approximately 35–40 µm but also more in some cases; Table 1). The longitudinally striated hairs are usually more or less straight and gradually taper toward a sharply pointed tip. The base of C2 is approximately 1.5 µm wide and is inserted in a well developed socket with an inner diameter up to 2.1 µm (Fig. 2B–G). No terminal pore is visible. These sensilla are present on all flagellomeres (A3–A9). They are generally located on the proximal half of each flagellomere but are more scattered on the distal half. On the first flagellomere (A3), they are essentially the only sensilla except for the conspicuous C1, with the other types of sensilla being very rare there. In all the studied species, sensilla C2 are the longest in female apterons compared with male apterons and all dealates. In *S. cervicornis*, the male apteron has longer C2 than the male dealate do (Table 1).

Because no terminal pore or wall pores were observed on the hair surface of sensilla C2, they may be classified among the aporous sensilla with flexible sockets, which are tactile mechanoreceptors^34^. Slifer and Sekhon^24^ called these sensilla tactile hairs (Tables 6, S11) and noted that they are unaffected by a 0.5% solution of crystal violet applied externally and that each such sensillum presumably contains a dendrite from a single neuron. While this type of sensilla forms fewer than 20% of all sensilla in *Spiralizoros cervicornis* and *Spermozoros weiweii*, it forms approximately 22–26% of all sensilla in apterons of *Spiralizoros* sp. and even slightly more than 30% in the female dealate of the same species (Table 5).

Sensilla chaetica C3 are present only in the apterons of both sexes of *Spermozoros weiweii* (Fig. 2E). These sensilla differ from sensilla chaetica C1 and C2 in their stocky appearance and can be easily recognized in SEM because of their lighter coloration. A single sensillum C3 is located at mid-length (A3; Fig. 2E) or at approximately two-thirds of each flagellomere. Sensilla C3 are semierect, at an angle of 45° from the antennal integument, and they are directed toward the distal end. The length of the sensilla C3 varies from 25.0 to 56.3 µm. They are widely inserted into a socket (with average internal and external diameters of approximately 6.5 and 8.2 µm, respectively) and have a dozen of longitudinal striae. The blunt-tipped hairs maintain their basal diameter for two-thirds of their length and then narrow toward the apex, with the terminal part twisted and with the truncate tip likely with a pore. The presence of a terminal pore is plausible but the uniporous character of these sensilla must be confirmed by future studies. Here, we tentatively assign a contact chemoreceptive function to sensilla chaetica C3 of *S. weiweii*.

#### Sensilla trichodea (T)

Sensilla trichodea are elongated, thin-walled hairs with narrowly rounded apices. In all the studied zorapterans, sensilla trichodea are always more numerous and generally slightly shorter and visibly narrower than sensilla chaetica C2 (Fig. 2C,F,G; Table 1). In most of the studied specimens, sensilla trichodea are usually about 30 µm long but are approximately twice longer in the female apteron of *Spiralizoros cervicornis* (Table 1). Owing to their very thin walls (already reported by Slifer and Sekhon^24^) in combination with dehydration in SEM, they were partly distorted in some cases, being bent and folded in half. In most of the cases, the proximal half was regularly slender, whereas the distal half was more abruptly attenuated toward the apex. Sensilla trichodea are distributed quite regularly and in high numbers all over the surface of flagellomeres 2–7 (A4–A9). They are also present on the first flagellomere (A3) but in much lower numbers, except for the male dealate of *S. cervicornis*, which lacks T on A3 (Tables 2, 3, 4, S3–S10). Sensilla trichodea are called "long, slender thin-walled chemoreceptors" by Slifer and Sekhon^24^ (Tables 6, S11). According to those authors, all thin-walled chemoreceptors (i.e., sensilla trichodea and sensilla basiconica 1 and 2) are very numerous on antennomeres A4–A9, but none occur on the first flagellomere (A3) in *Usazoros hubbardi*. There are up to seven sensilla trichodea on A3 in *Spiralizoros* spp. and up to 11 in *Spermozoros weiweii* (Tables 2, 3, 4, S3–S10). Slifer and Sekhon^24^ reported that these sensilla have multiple small pores in their walls. All electron microscopy and electrophysiology studies revealed that sensilla with a multiporous wall have an olfactory function^35,36^. Sensilla trichodea constitute 43.4–44.5% of all antennal sensilla in *Spiralizoros* sp., 17.4–27.7% in *S. cervicornis*, and 30.2–39.7% in *Spermozoros weiweii* (Table 5).

#### Sensilla basiconica (B1–3)

These sensilla are usually distinctly shorter than sensilla chaetica and trichodea. Two main subtypes (B1 and B2) are present in all the here-examined species, whereas the third subtype (B3) is present only in *Spermozoros*. Sensilla basiconica are called "short, slender thin-walled chemoreceptors" (B1) and "wide thin-walled chemoreceptors" (B2) by Slifer and Sekhon^24^ (Tables 6, S11). According to those authors, both main subtypes of sensilla basiconica have multiple small pores in their walls which suggests they have an olfactory function^35,36^. These chemoreceptors are very numerous on antennomeres A4–A9, but none occur on the first flagellomere (F1, = A3) in *Usazoros hubbardi*^24^. In both genera examined by us, i.e., *Spermozoros* and *Spiralizoros*, we observed sensilla basiconica B1 and B2 (either one subtype or both) on A3 of some specimens but always in much lower numbers than on A4–A9 (Tables 2, 3, 4, S3–S10). Sensilla basiconica form 8.4 to 12.6% of all antennal sensilla in *Spiralizoros* sp., 36.2–52.3% in *S. cervicornis*, and 31.4–38.0% in *Spermozoros weiweii* (Table 5).

Sensilla basiconica B1 are slender and relatively short hairs inserted in a socket. They have narrowly rounded apices, and their mean lengths range from 12.1 to 21.3 µm (mean widths of 0.5–0.7 µm), depending on the species and morphological form. They are usually distinctly shorter than sensilla trichodea, often being approximately half of their length (Fig. 2C–E, G; Table 1). Especially in the distal parts of the flagellomeres, they can be easily identified, because their lengths are similar to those of sensilla basiconica B2 (which occur mainly in distal regions of flagellomeres and are easily recognizable). Sensilla basiconica B1, particularly those on the sides of flagellomeres, were in some cases distorted due to the dehydration process for SEM, as they have very thin walls. These sensilla are rare or absent on the first flagellomere (A3) but are present in greater numbers on both faces of flagellomeres 2–9. There are marked differences in the number of B1 between the two studied *Spiralizoros* species. While *Spiralizoros* sp. has no B1 on the first flagellomere and only 5–20 B1 on each of the flagellomeres 2–7, *S. cervicornis* has 1–4 B1 on the first flagellomere and 24–81 B1 on each of the following flagellomeres. Interestingly, *Spermozoros weiweii* is in this aspect more similar to the latter, having 1–8 B1 on the first flagellomere and 22–85 B1 on each of the flagellomeres 2–7 (Tables 2, 3, 4, S3–S10). Similarly, B1 constitute only up to 10.6% of all sensilla in *Spiralizoros* sp., while they form 35.4 to 42.4% of all sensilla in *S. cervicornis* and 25.5–34.0% in *Spermozoros weiweii* (Table 5).

Sensilla basiconica 2 are easily recognizable due to their more or less tongue-like shape with approximately the same width from the base toward the subapical part (Fig. 2G,H). These sensilla are inserted in a nonflexible socket. Their mean lengths and widths are very similar in both sexes and morphological forms of *Spiralizoros* sp. (18.1–18.2 µm and 2.5 µm, respectively) and both sexes of *Spermozoros weiweii* (14.3–14.5 µm and 2.3–2.4 µm, respectively), and variable in *Spiralizoros cervicornis* (10.5–20.0 µm and 2.1–2.5 µm, respectively) (Table 1). In addition to their typical shape, sensilla basiconica 2 are well characterized by their number and distribution. Sensilla B2 occur mainly in distal regions of flagellomeres. They are present in small numbers on both faces of flagellomeres 2–7 (A4–A9) and are rare or absent on the first flagellomere. In *Spiralizoros* sp., they start either from A3 (female dealate), A4 (female apteron) or A6 (male apteron), with each flagellomere bearing 1–7 SB2. In *Spiralizoros cervicornis*, they start either from A3 (male apteron and dealate) or A7 (female apteron), with each flagellomere bearing 1–23 B2. While sensilla B2 were rather low in number in *S. cervicornis* apterons (up to six if we did not count the last antennomere of the male apteron), they were more numerous in the conspecific male dealate, with flagellomeres 2–7 each bearing 10–23 B2. In *Spermozoros weiweii*, sensilla B2 start either from A3 (female apteron) or A4 (male apteron), with each flagellomere 2–7 bearing 3–15 B2 (Tables 2–4, S3–S10). The male apteron had mostly more sensilla per flagellomere 2–7 (5–15) than the female apteron (3–8). The percentage of B2 from all sensilla on zorapteran flagellomeres was usually very low and reached between 0.8 and 4.8% in all specimens but one, which was the male dealate of *Spiralizoros cervicornis* with 9.9% (Table 5). It would be interesting to examine winged males or dealates of other species if they also have relatively more sensilla B2.

Sensilla basiconica 3 were found only on the dorsal face of the antenna in both the male and female apterons of *Spermozoros weiweii* (Fig. 2G). If present, they are typically located on the distal part of the flagellomere, among sensilla basiconica B1 (always in the male apteron; Fig. 2G), but in the female apteron, they are sometimes also located on the proximal half. These sensilla differ from sensilla basiconica 1 in their stockier appearance and blunt tips; they are approximately as long as or slightly shorter than sensilla B1 but with the basal width of sensilla trichodea (Table 1). No pores were visible via SEM. In the male apteron, these sensilla start from F3 (A5), with each flagellomere 3–7 bearing 1–3 B3 (11 in total). In the female apteron, they start from F4 (A6), with each flagellomere 4–7 bearing 2–3 B3 (nine in total). Although we failed to observe the pores in the walls of sensilla B3, an olfactory function is hypothesized for these sensilla in *Spermozoros weiweii*. However, we cannot rule out the gustatory function without the transmission electron microscopic studies.

Sensilla with presumed olfactory function in Zoraptera are sensilla trichodea and basiconica which together constitute about 53–71% of all sensilla on A3–A9. More specifically, they form 52.9 to 57.1% of all sensilla on flagellomeres in *Spiralizoros* sp., 63.9–69.7% in *S. cervicornis*, and 68.2–71.1% in *Spermozoros weiweii*. There were no significant differences between the sexes or morphological forms of the same species. Both sensilla trichodea and basiconica in all the studied Zoraptera vary greatly in length and witdth (Table 1). Slifer and Sekhon^24^ already reported series of long and short chemoreceptors in *Usazoros hubbardi* (on 14 examined antennae) which show variation of lengths without any functional meaning. The olfactory sensilla in insects usually enable to detect and identify volatile compounds for foraging, predator or toxic food avoidance, finding mating partners via pheromones, and locating oviposition sites^37–39^. The specific functions of olfactory sensilla in Zoraptera need to be researched in the future.

#### Sensilla styloconica (St)

Sensilla styloconica are present only in the male and female apteron of *Spermozoros weiweii*. They comprise a basal stylus, which is 1.5 µm long and 1.5 µm wide, and a sensory cone, which is 2.0 µm long and 1.1 µm wide at the base (Fig. 2D,G). The sensillum base is widely inserted into a socket, which is bordered by a thick circular bulge with internal and external diameters of approximately 2.5 µm and 6.5 µm, respectively. No terminal pore or wall pores were observed. A single sensillum is usually situated medially on the extreme distal part on the dorsal face of many flagellomeres. In the male apteron, distal flagellomere A9 bears three sensilla styloconica: one at one-third of its length and two at the apex. In the female apteron, only a single sensillum styloconicum was identified on A9.

The shape, distribution, and number of sensilla styloconica in Zoraptera are similar to the aporous sensilla styloconica present on flagellomeres of Lepidoptera^27^. Most probably they have a thermo-hygroreceptive function as suggested by Steinbrecht^40^. It is interesting that this sensillum type was reported for all studied species of Lepidoptera; however, it is present only in a single species of Zoraptera. We would expect that sensilla styloconica are present in all species and that in both examined species of *Spiralizoros* they are located at about the same place as in *Spermozoros* (as in other insect groups) but we failed to observe any sensilla styloconica there. More lineages of both subfamilies should be investigated in order to confirm the taxonomic importance of these sensilla in Zoraptera.

### Comparison of antennal sensilla within Zoraptera

The antennae of all known recent Zoraptera comprise of nine antennomeres in adult stage (nymphs have eight antennomeres), while the fossil adult zorapterans have either nine or eight antennomeres^10,11^. Most species with eight antennomeres were originally classified in the extinct *Octozoros* Engel, 2003 which was considered a subgenus of *Zorotypus* and is known exclusively from the mid-Cretaceous amber of northern Myanmar^18,41^. A single species was described in the genus *Palaeospinosus* Kaddumi, 2005 from the Lower Cretaceous Jordanian amber^42^ and later transferred to *Octozoros* by Engel^4^. Recently, Kočárek et al.^10^ critically reviewed the fossil zorapterans and included all known species with eight antennomeres into three genera (including above-mentioned *Octozoros* and *Palaeospinosus*) within Spiralizoridae: Latinozorinae.

Regarding the extant Zoraptera, here we examined antennae of the representatives of two genera, *Spermozoros* and *Spiralizoros*, and the third genus, *Usazoros*, was studied already in 1978. *Spermozoros* and *Usazoros* belong to the family Zorotypidae, whereas *Spiralizoros* to Spiralizoridae. The general shape of antennae does not differ between the two families, and the same can be concluded for the genera, species, sexes, and morphological forms. However, there are some differences in the relative lengths of basal antennomeres between the genera in Zoraptera^11^. Our results are consistent with diagnoses of *Spermozoros* and *Spiralizoros*^11^ as well as with diagnoses of *Spermozoros weiweii*^25^ and *Spiralizoros cervicornis*^26^ (Table S2).

One would expect that *Spermozoros* and *Usazoros*, as representatives of the same family, will have more similarities to each other in their antennal sensillar equipment than any of them to *Spiralizoros*. However, here we observed that *Spiralizoros* has the same types of sensilla as previously reported for *Usazoros*^24^, and *Spermozoros* has three additional (sub)types (Tables 6, S11). It should be noted, however, that only one specimen per sex and form (apteron, dealate) was used in our study due to the extreme rarity of examined specimens. Consequently, we cannot rule out that the observed variation between morphs and sexes could be attributed to individual variation. Slifer and Sekhon^24^ identified five types of sensory receptors on the antennal flagellum of wingless forms of *Usazoros hubbardi*, i.e., tactile hairs (1 type: A), thick-walled chemoreceptors (1 type: B), and thin-walled chemoreceptors (3 types: C, D, E). According to the terminology by Zacharuk^34^, those sensilla types can be classified as follows: A as aporous sensilla chaetica, B as uniporous sensilla chaetica, and C–E as multiporous sensilla trichodea or basiconica. All five types are present in all examined specimens of *Spiralizoros* and *Spermozoros*; however, the latter has three additional (sub)types (Tables 6, S11). We cannot exclude the possibility that in 1978, not having techniques available in the twenty-first century, Slifer and Sekhon^24^ overlooked some types of sensilla which we report here for *Spermozoros*. Therefore, further study including more genera, mainly *Usazoros*, will help to clarify this situation.

Interestingly, *Spiralizoros cervicornis* was in some aspects more similar to *Spermozoros weiweii* than to the second species of *Spiralizoros*. The most striking example is the ratio of all sensilla chaetica to all other sensilla on flagellomeres. Sensilla chaetica constitute 42.9–47.1% of all antennal sensilla on flagellomeres in *Spiralizoros* sp., whereas it is only 30.3–36.1% in *Spiralizoros cervicornis* and 28.1–31.2% in *Spermozoros weiweii*. Consequently, sensilla trichodea and basiconica, i.e., those with the presumed olfactory function, constitute only 52.9–57.1% of all sensilla on flagellomeres in *Spiralizoros* sp., whereas it is 63.9–69.7% in *S. cervicornis* and 68.2–71.1% in *Spermozoros weiweii* (Table 5). *Spiralizoros cervicornis* is more similar to *Spermozoros weiweii* also in the organization and numbers of sensilla basiconica 1, as well as in their proportion of all sensilla; while sensilla B1 constitute only up to 10.6% of all sensilla in *Spiralizoros* sp., it is 35.4–42.4% in *S. cervicornis* and 25.5–34.0% in *Spermozoros weiweii* (Table 5). Regarding the differences between male and female apterons or apterons and dealates, again we did not find almost any taxonomic pattern. The difference between the examined genera is in sensilla trichodea; while female apterons of *Spiralizoros* spp. had relatively slightly more sensilla trichodea than their male counterparts, in *Spermozoros weiweii* the male apteron had significantly more sensilla trichodea than female apteron (39.7% versus 30.2%; Table 5). However, female apterons of *Spiralizoros cervicornis* and *Spermozoros weiweii* had relatively slightly more sensilla chaetica and less olfactory sensilla (trichodea and basiconica) than their male counterparts (difference approximately 3%), whereas female apterons of *Spiralizoros* sp. had it vice versa but with even less difference between sexes (Table 5).

The morphological differences between the related species, including the differences in antennal sensilla, could usually be explained by different ecological and behavioral aspects of the given groups; however, since our knowledge on biology and ecology of Zoraptera is limited and they all live in very similar habitats, it is difficult even to speculate about the differences between the examined lineages. We hypothesized there are significant differences in sensillar equipment (especially in the proportion of sensilla with olfactory function) of apterons and dealates (i.e., alates that shed their wings) within a single species as alates have to fly and search for the optimal habitat, i.e., a rotten log in a specific stage of decomposition. However, we did not find any pattern common for both species in which we had both morphological forms available. Therefore, dealates (or alates) of both sexes should be investigated for as many species as possible and compared to respective apterons in order to have more conclusive results.

Slifer and Sekhon^24^ reported the presence of an “apical organ” on the ultimate antennomere of *Usazoros hubbardi*. Here, we observed at the apices of ultimate antennomeres elongate sensillum which were significantly longer than the surrounding sensilla. For example, in *Spiralizoros cervicornis*, the male dealate had there a sensillum which resembles extremely long sensillum basiconicum 2 (almost 30 µm long, whereas other SB2 on A9 measured only 12.5–15.8 µm), whereas male and female apterons had the apex of A9 extended by structure similar to sensillum chaeticum of subtype 2 or 3. These structures should be investigated using SEM and TEM in future studies when more material is available.

### Comparison of antennal sensilla of Zoraptera with related groups

The phylogenetic placement of Zoraptera has always been a matter of debate^14^. Recent phylogenomic analyses constantly recovered Zoraptera as a part of Polyneoptera and sister to Dermaptera^6,8,15–17^ (the relationship at least partly supported also by morphology^43^), although Tihelka et al.^9^ found Zoraptera sister to all remaining Polyneoptera. Slifer and Sekhon^24^ pointed out the similarities in antennal morphology between *Usazoros hubbardi* and termites. However, these two insect groups are only distantly related, as termites are part of Dictyoptera (Blattodea and Mantodea)^15,44^. The antennal sensilla in Dermaptera were studied by Slifer^45^, Al-Dosary et al.^46^, and Faucheux^47^. We can conclude that the sensillar equipment of Zoraptera and Dermaptera is generally similar, although in the latter, no sensilla trichodea and styloconica were reported, and, on the other hand, they have sensilla coeloconica which are missing in Zoraptera (Table S11). This comparison is, however, based on a very limited sampling. Greater taxonomic diversity should be sampled for future studies on the antennal micromorphology of these groups.

Although, as we noted above, the presumed “apical organs” of Zoraptera should be investigated in future studies, it is worth mentioniong that such structures are present in other polyneopteran insects like Mantodea^48,49^ and Orthoptera^50^. However, they can also be found in some other insects, like antlions (Neuroptera) (M. J. F., pers. observ.). Another interesting character in Polyneoptera is the number and organization of sensilla campaniformia on pedicel^47^. The arrangement of sensilla Ca in two circles around the apex of pedicel is characteristic for the orthopteroid complex^50–52^ but it was also reported for Blattodea^53^, Mantodea^49^, and Phasmatodea^54^. On the other hand, the circular arrangement of sensilla Ca is not present in Isoptera^55^ and Zoraptera. A single sensillum campaniformium located at the distal edge of pedicel near the joint with A3 in zorapterans is similar as in various other insects, like Psocoptera^56^, Lepidoptera^27^, and Diptera^57^.

## Conclusions

In this preliminary study, we compared antennal sensilla of three species belonging to two genera, *Spiralizoros* and *Spermozoros*, each representing one of two currently recognized families in Zoraptera, Spiralizoridae and Zorotypidae, respectively. In all eight examined specimens, we identified the same types of sensilla as Slifer and Sekhon^24^ did previously for *Usazoros hubbardi*, and three additional (sub)types were found in *Spermozoros*. We found interesting differences between two species of *Spiralizoros* in the relative numbers of sensilla chaetica and olfactory sensilla (trichodea and basiconica). Since we did not find any pattern common for apterons and dealates in two species for which both morphological forms were available, more robust data is needed for more conclusive results. As this study is just preliminary and serves as the first step toward more detailed research on antennal morphology within Zoraptera, representatives of all genera should be examined using SEM, including yet unstudied subfamilies Zorotypinae (Zorotypidae) and Latinozorinae (Spiralizoridae). Additionally, almost half century old results by Slifer and Sekhon^24^ on antennal sensilla of *Usazoros hubbardi* should be re-investigated using modern methods. Although we here proposed probable functions of studied sensilla based on external morphology, more detailed studies using TEM should be carried out to confirm our hypotheses and mainly to investigate if sensilla chaetica C1 are contact chemoreceptors or tactile mechanoreceptors. Since Zoraptera are hypothesized to be closely related to Dermaptera in some recent studies^6,8,15–17^, the comparison of antennal sensillar equipment between these two orders would be desirable. The antennal sensilla of Dermaptera were properly figured and examined by the means of SEM only for the common European species *Forficula auricularia* Linnaeus, 1758^46^; therefore, wider diversity of Dermaptera across their tree of life should be included into the SEM studies.

## Material and methods

The classification of Zoraptera is based on the studies by Kočárek et al.^10,11^. As zorapterans are dimorphic insects, we followed Engel^4^ in using the “apteron” for an individual of the blind and wingless morph, the “alate” for a fully winged individual with developed compound eyes and ocelli, and the “dealate” for an alate that shed its wings. Our study is based on adult specimens. We examined the morphology, numbers, and distribution of antennal sensilla in apterons of both sexes for *Spiralizoros cervicornis* from Brunei Darussalam, Ulu Temburong National park (collected in 2015; ID number 57 in Kaláb et al.^2^), *Spiralizoros* sp. (both Spiralizoridae: Spiralizorinae) from Malaysia, Sabah, Kinasaraban env. (collected in 2009; ID number 27^2^), and *Spermozoros weiweii* (Zorotypidae: Spermozorinae) from Brunei Darussalam, Ulu Temburong National park (collected in 2015; ID number 534^2^) (Table S1). Furthermore, we also compared their sensillar equipment with those of a dealate male of *Spiralizoros cervicornis* (ID number 53^2^) and a dealate female of *Spiralizoros* sp. (ID number 27^2^). An aspirator was used to collect the specimens from under the bark of decaying trees. All examined specimens were stored in 96% ethanol. In this initial phase of the antennal micromorphology research on Zoraptera, only one specimen per sex and form (apteron, dealate) were used due to the extreme rarity of examined specimens.

For scanning electron microscopy (SEM), the entire insects were air-dried on a filter paper. To achieve the best results, the insects were placed laterally on an alcohol-saturated filter paper because the lightly sclerotized, soft antennae spread out and stick on a filter paper, ideally one by its ventral surface, and the other by the dorsal surface. The insects were mounted on specimen holders with a scotch double face. After coating with a layer of carbon (around 25 nm thick) in a carbon thread evaporation device BAL-TEC CED 030, they were examined using a Jeol JSM 5800 LV scanning electron microscope at 12 kV.

The antennal length was measured between the base of scape and the tip of ultimate antennomere. For the counts of sensilla, we considered four faces: the dorsal and the ventral faces on which the bases of sensilla were visible, and two lateral faces where the bases were usually not visible. All sensilla were counted manually based on SEM observations. For each sex and form, the average length and basal width of various types of antennal sensilla were calculated based on 20 measurements taken from SEM micrographs. The observed sensilla were classified according to their external morphology, distribution, and presence or absence of pores, following Altner and Prillinger, Zacharuk^34, 59^, and Faucheux. We compared our results with those of Slifer and Sekhon who studied the micromorphology of antennal flagellum of *Usazoros hubbardi* from Florida, USA.

## Electronic supplementary material

Below is the link to the electronic supplementary material.

Supplementary Material 1

## Data Availability

All data generated or analysed during this study are included in this published article.
