# Supplementary material for: An initial comparative study on the antennal morphology of Zoraptera (Insecta) with special reference to the sensilla
Source: Sci Rep. 2025 Jul 5;15:24057. doi: 10.1038/s41598-025-08212-x (PMC12228702; doi:10.1038/s41598-025-08212-x)
Supplement: Supplementary file 1 — Supplementary Material 1 [file 41598_2025_8212_MOESM1_ESM.pdf]

Supplementary Information

An initial conmparative study on the antennal morphology of Zoraptera (Insecta), with special reference to the sensilla

Michel J. Faucheux[1,*], Gabriela Packova[2], Petr Kočárek[3] & Robin Kundrata[2,*]

[1]Laboratoire d'Endocrinologie des Insectes Sociaux, Faculté des Sciences et des Techniques, 2 rue de la Houssinière, B.P. 92208, 44322, Nantes Cedex 03, France
[2]Department of Zoology, Faculty of Science, Palacky University, 17. listopadu 50, 77146, Olomouc, Czech Republic
[3]Department of Biology and Ecology, Faculty of Science, University of Ostrava, Chitussiho 10, 71000, Ostrava 2, Czech Republic
*Corresponding authors. E-mails: robin.kundrata@upol.cz (R.K.), faucheux.michel@free.fr (M.J.F.).

List of Supplementary Tables

Table S1. List of examined specimens.

Table S2. Length and width of antennomeres 1–9 of studied specimens of Zoraptera.

Table S3. Numbers of sensilla on antennae of *Spiralizoros* sp., male apteron.

Table S4. Numbers of sensilla on antennae of *Spiralizoros* sp., female apteron.

Table S5. Numbers of sensilla on antennae of *Spiralizoros* sp., female dealate.

Table S6. Numbers of sensilla on antennae of *Spiralizoros cervicornis*, male apteron.

Table S7. Numbers of sensilla on antennae of *Spiralizoros cervicornis*, female apteron.

Table S8. Numbers of sensilla on antennae of *Spiralizoros cervicornis*, male dealate.

Table S9. Numbers of sensilla on antennae of *Spermozoros weiweii*, male apteron.

Table S10. Numbers of sensilla on antennae of *Spermozoros weiweii*, female apteron.

Table S11. Antennal sensillum (sub)types in Zoraptera and Dermaptera.

Table S1. List of examined specimens. "Kaláb ID" refers to the ID number within the Zoraptera dataset in Kaláb et al. (in press).

Genus/Species	Sex	Form	Collecting data	Kaláb ID
Spiralizoridae: Spiralizorinae				
Spiralizoros sp.	male	apteron	Malaysia, Kinasaraban env., 5°58'10.1"N, 116°33'27.5"E, 15.i.2009, Ř. Fafejt leg.	27
Spiralizoros sp.	female	apteron	Malaysia, Kinasaraban env., 5°58'10.1"N, 116°33'27.5"E, 15.i.2009, Ř. Fafejt leg.	27
Spiralizoros sp.	female	dealate	Malaysia, Kinasaraban env., 5°58'10.1"N, 116°33'27.5"E, 15.i.2009, Ř. Fafejt leg.	27
S. cervicornis	male	apteron	Brunei Darussalam, Ulu Temburong NP, Sungai Serulu, 4°33'51.6"N, 115°8'58.4"E, 12.-19.ii.2015, P. Kočárek & I. Horká leg.	57
S. cervicornis	female	apteron	Brunei Darussalam, Ulu Temburong NP, Sungai Serulu, 4°33'51.6"N, 115°8'58.4"E, 12.–19.ii.2015, P. Kočárek & I. Horká leg.	57
S. cervicornis	male	dealate	Brunei Darussalam, Ulu Temburong NP, Eshton trail, 4°32'42.3"N, 115°8'45.6"E, 12.–19.ii.2015, P. Kočárek & I. Horká leg.	53
Zorotypidae: Spermozorinae				
Spermozoros weiweii	male	apteron	Brunei Darussalam, Ulu Temburong NP, Sungai Esu stream, 04°32'14.1"N, 115°9'47.1"E, 12.–19.ii.2015, P. Kočárek & I. Horká leg.	534
Spermozoros weiweii	female	apteron	Brunei Darussalam, Ulu Temburong NP, Sungai Esu stream, 04°32'14.1"N, 115°9'47.1"E, 12.–19.ii.2015, P. Kočárek & I. Horká leg.	534

Table S2. Length (upper value) and width (measured medially; lower value) of antennomeres 1–9 of studied specimens of Zoraptera (in µm). M = male, F = female, apt = apteron, dea = dealate.

Antennomere		A1	A2	A3	A4	A5	A6	A7	A8	A9	Total
Spiralizoros sp.	M apt	103.4	64.3	119.5	149.4	195.3	172.4	172.4	161.3	174.2	1319.2
		80.7	50.6	62.1	73.5	69.2	64.3	64.3	58.1	45.2	
	F apt	141.2	74.1	129.4	141.2	177.2	181.2	169.4	164.7	192.9	1371.3
		75.3	67.7	58.8	65.9	68.2	65.9	70.6	68.3	51.7	
	F dea	175.0	85.0	170.1	229.8	253.7	232.8	208.9	200.0	200.0	1755.3
		85.6	50.4	59.7	74.6	74.6	74.6	74.6	65.6	38.9	
Spermozoros weiweii	M apt	144.4	62.2	112.2	187.5	205.0	200.0	162.5	137.5	162.5	1373.8
		71.1	44.4	66.6	70.0	70.0	67.5	62.5	75.0	33.0	
	F apt	133.3	50.8	104.8	152.4	208.7	200.0	205.0	192.5	200.0	1447.5
		79.3	44.4	66.6	69.8	71.4	62.8	65.0	62.5	42.4	
Spiralizoros cervicornis	M apt	153.8	65.4	134.6	161.5	307.7	184.6	172.1	134.6	192.3	1406.6
		76.8	53.6	53.6	61.5	61.5	61.5	57.7	57.7	53.8	
	F apt	149.7	55.5	148.2	185.2	214.8	293.7	192.6	166.6	185.2	1492.5
		96.3	62.9	66.6	66.6	70.4	62.9	59.2	62.9	74.1	
	M dea	125.0	69.4	111.1	173.6	215.3	194.4	189.5	159.7	173.6	1402.6
		76.4	48.6	55.5	59.8	59.8	69.4	62.5	52.8	41.7	

Table S3. Numbers of sensilla on antennae of *Spiralizoros* sp., male apteron. A1–A9 = antennomeres 1–9; df, vf, lf = dorsal, ventral, and lateral face; af - all faces together.

Sensilla		A1	A2	A3	A4	A5	A6	A7	A8	A9	Total
Chaetica C1	df	7	3	3	3	4	7	5	4	3	39
	vf	10	4	5	5	3	4	4	5	5	45
	lf	10	5	5	3	7	6	7	3	6	52
	**af**	**27**	**12**	**13**	**11**	**14**	**17**	**16**	**12**	**14**	**136**
Chaetica C2	df	-	-	12	4	7	13	8	11	13	68
	vf	-	-	5	3	9	7	5	9	8	46
	lf	-	-	14	8	14	18	13	14	9	90
	**af**	**-**	**-**	**31**	**15**	**30**	**38**	**26**	**34**	**30**	**204**
Trichodea	df	-	-	4	11	11	12	16	11	13	78
	vf	-	-	3	16	19	27	25	15	17	122
	lf	-	-	5	13	22	18	27	29	21	135
	**af**	**-**	**-**	**12**	**40**	**52**	**57**	**68**	**55**	**51**	**335**
Basiconica B1	df	-	-	-	5	2	7	4	3	4	25
	vf	-	-	-	4	5	8	5	3	2	27
	lf	-	-	-	7	4	5	6	5	3	30
	**af**	**-**	**-**	**-**	**16**	**11**	**20**	**15**	**11**	**9**	**82**
Basiconica B2	df	-	-	-	-	-	-	2	1	1	4
	vf	-	-	-	-	-	2	3	1	2	8
	lf	-	-	-	-	-	1	2	-	-	3
	**af**	-	-	-	-	-	**3**	**7**	**2**	**3**	**15**
Total		**27**	**12**	**56**	**82**	**107**	**135**	**132**	**114**	**107**	**772**

Table S4. Numbers of sensilla on antennae of *Spiralizoros* sp., female apteron. A1–A9 = antennomeres 1–9; df, vf, lf = dorsal, ventral, and lateral face; af - all faces together.

Sensilla		A1	A2	A3	A4	A5	A6	A7	A8	A9	Total
Chaetica C1	df	6	5	4	4	3	2	2	2	3	31
	vf	7	8	5	2	6	4	6	5	4	47
	lf	12	14	7	4	3	4	1	6	5	56
	**af**	**25**	**27**	**16**	**10**	**12**	**10**	**9**	**13**	**12**	**134**
Chaetica C2	df	-	-	6	6	7	4	5	2	6	36
	vf	-	-	5	8	6	10	11	6	9	55
	lf	-	-	3	3	8	9	10	4	10	47
	**af**	**-**	**-**	**14**	**23**	**21**	**23**	**26**	**12**	**25**	**144**
Trichodea	df	-	-	2	13	12	12	21	15	18	93
	vf	-	-	-	10	13	20	12	18	14	87
	lf	-	-	2	14	18	23	28	10	14	109
	**af**	**-**	**-**	**4**	**37**	**43**	**55**	**61**	**43**	**46**	**289**
Basiconica B1	df	-	-	-	6	6	5	3	1	3	24
	vf	-	-	-	7	3	3	2	1	2	18
	lf	-	-	-	4	1	2	-	6	5	18
	**af**	**-**	**-**	**-**	**17**	**10**	**10**	**5**	**8**	**10**	**60**
Basiconica B2	df	-	-	-	2	-	-	-	-	-	2
	vf	-	-	-	3	3	3	2	3	2	16
	lf	-	-	-	-	-	2	2	-	-	4
	**af**	-	-	-	**5**	**3**	**5**	**4**	**3**	**2**	**22**
Total		**25**	**27**	**34**	**92**	**89**	**103**	**105**	**79**	**95**	**649**

Table S5. Numbers of sensilla on antennae of *Spiralizoros* sp., female dealate. A1–A9 = antennomeres 1–9; df, vf, lf = dorsal, ventral, and lateral face; af - all faces together.

Sensilla		A1	A2	A3	A4	A5	A6	A7	A8	A9	Total
Chaetica C1	df	10	4	2	3	4	5	3	3	2	36
	vf	12	7	1	3	3	3	4	3	2	38
	lf	17	6	8	5	4	4	6	8	6	64
	**af**	**39**	**17**	**11**	**11**	**11**	**12**	**13**	**14**	**10**	**138**
Chaetica C2	df	-	-	4	16	13	10	9	9	8	69
	vf	-	-	5	4	10	9	9	8	7	52
	lf	-	-	14	28	22	17	20	14	17	132
	**af**	**-**	**-**	**23**	**48**	**45**	**36**	**38**	**31**	**32**	**253**
Trichodea	df	-	-	1	15	19	30	37	22	26	150
	vf	-	-	-	10	15	17	20	23	12	97
	lf	-	-	-	12	18	21	23	29	19	122
	**af**	**-**	**-**	**1**	**37**	**52**	**68**	**80**	**74**	**57**	**369**
Basiconica B1	df	-	-	-	3	1	1	2	3	3	13
	vf	-	-	-	2	4	2	2	5	1	16
	lf	-	-	-	6	3	6	4	2	3	24
	**af**	**-**	**-**	**-**	**11**	**8**	**9**	**8**	**10**	**7**	**53**
Basiconica B2	df	-	-	1	-	-	-	1	3	2	7
	vf	-	-	1	1	3	2	-	-	-	7
	lf	-	-	-	2	1	-	-	-	-	3
	**af**	-	-	**2**	**3**	**4**	**2**	**1**	**3**	**2**	**17**
Total		**39**	**17**	**37**	**110**	**120**	**127**	**140**	**132**	**108**	**830**

Table S6. Numbers of sensilla on antennae of *Spiralizoros cervicornis*, male apteron. A1–A9 = antennomeres 1–9; df, vf, lf = dorsal, ventral, and lateral face; af - all faces together.

Sensilla		A1	A2	A3	A4	A5	A6	A7	A8	A9	Total
Chaetica C1	df	10	6	3	4	4	6	4	5	5	47
	vf	18	7	8	4	6	5	4	4	5	61
	lf	7	3	2	6	7	9	8	9	9	60
	**af**	**35**	**16**	**13**	**14**	**17**	**20**	**16**	**18**	**19**	**168**
Chaetica C2	df	-	-	3	4	7	5	6	5	4	34
	vf	-	-	6	10	10	12	9	7	8	62
	lf	-	-	2	8	12	4	4	10	2	42
	**af**	**-**	**-**	**11**	**22**	**29**	**21**	**19**	**22**	**14**	**138**
Trichodea	df	-	-	2	8	9	15	9	19	7	69
	vf	-	-	1	11	7	18	12	10	13	72
	lf	-	-	3	14	26	11	15	11	11	91
	**af**	**-**	**-**	**6**	**33**	**42**	**44**	**36**	**40**	**31**	**232**
Basiconica B1	df	-	-	-	9	15	21	15	13	14	87
	vf	-	-	-	12	28	27	13	17	25	122
	lf	-	-	2	17	24	25	24	22	42	156
	**af**	**-**	**-**	**2**	**38**	**67**	**73**	**52**	**52**	**81**	**365**
Basiconica B2	df	-	-	-	1	1	2	1	2	5	12
	vf	-	-	-	1	1	2	2	4	8	18
	lf	-	-	1	-	-	-	2	-	4	7
	**af**	-	-	**1**	**2**	**2**	**4**	**5**	**6**	**17**	**37**
Total		**35**	**16**	**33**	**109**	**157**	**162**	**128**	**138**	**162**	**940**

Table S7. Numbers of sensilla on antennae of *Spiralizoros cervicornis*, female apteron. A1–A9 = antennomeres 1–9; df, vf, lf = dorsal, ventral, and lateral face; af - all faces together.

Sensilla		A1	A2	A3	A4	A5	A6	A7	A8	A9	Total
Chaetica C1	df	2	2	3	3	11	6	4	4	4	39
	vf	6	2	5	10	2	10	8	6	8	57
	lf	5	3	7	2	4	2	2	3	5	33
	**af**	**13**	**7**	**15**	**15**	**17**	**18**	**14**	**13**	**17**	**129**
Chaetica C2	df	-	-	6	6	2	7	9	11	5	46
	vf	-	-	8	7	6	12	7	8	7	55
	lf	-	-	10	13	7	9	7	5	2	53
	**af**	**-**	**-**	**24**	**26**	**15**	**28**	**23**	**24**	**14**	**154**
Trichodea	df	-	-	-	7	4	8	7	25	19	70
	vf	-	-	2	12	7	10	8	6	14	59
	lf	-	-	5	9	21	18	12	13	10	88
	**af**	**-**	**-**	**7**	**28**	**32**	**36**	**27**	**44**	**43**	**217**
Basiconica B1	df	-	-	-	5	13	25	19	8	17	87
	vf	-	-	-	11	9	17	12	15	14	78
	lf	-	-	4	8	15	21	30	13	22	113
	**af**	**-**	**-**	**4**	**24**	**37**	**63**	**61**	**36**	**53**	**278**
Basiconica B2	df	-	-	-	-	-	-	-	-	2	2
	vf	-	-	-	-	-	-	-	-	-	-
	lf	-	-	-	-	-	-	1	1	2	4
	**af**	-	-	-	**-**	**-**	**-**	**1**	**1**	**4**	**6**
Total		**13**	**7**	**50**	**93**	**101**	**145**	**126**	**118**	**131**	**784**

Table S8. Numbers of sensilla on antennae of *Spiralizoros cervicornis*, male dealate. A1–A9 = antennomeres 1–9; df, vf, lf = dorsal, ventral, and lateral face; af - all faces together.

Sensilla		A1	A2	A3	A4	A5	A6	A7	A8	A9	Total
Chaetica C1	df	7	4	8	4	9	8	5	4	4	53
	vf	8	5	6	5	7	10	4	6	3	54
	lf	6	3	6	7	6	5	6	6	5	50
	**af**	**21**	**12**	**20**	**16**	**22**	**23**	**15**	**16**	**12**	**157**
Chaetica C2	df	1	-	1	3	3	7	7	7	3	32
	vf	-	-	2	5	6	6	9	5	2	35
	lf	-	-	4	6	7	3	7	5	7	39
	**af**	**1**	**-**	**7**	**14**	**16**	**16**	**23**	**17**	**12**	**106**
Trichodea	df	-	-	-	11	5	10	8	6	9	49
	vf	-	-	-	9	7	8	6	5	10	45
	lf	-	-	-	10	11	5	11	9	16	62
	**af**	**-**	**-**	**-**	**30**	**23**	**23**	**25**	**20**	**35**	**156**
Basiconica B1	df	-	-	-	16	25	30	21	19	12	123
	vf	-	-	-	12	18	26	23	24	15	118
	lf	-	-	1	23	32	13	15	17	20	121
	**af**	**-**	**-**	**1**	**51**	**75**	**69**	**59**	**60**	**47**	**362**
Basiconica B2	df	-	-	-	6	3	3	5	2	5	24
	vf	-	-	-	7	5	4	5	5	4	30
	lf	-	-	3	10	6	4	4	3	2	32
	**af**	-	-	**3**	**23**	**14**	**11**	**14**	**10**	**11**	**86**
Total		**22**	**12**	**31**	**134**	**150**	**142**	**136**	**123**	**117**	**867**

Table S9. Numbers of sensilla on antennae of *Spermozoros weiweii*, male apteron. A1–A9 = antennomeres 1–9; df, vf, lf = dorsal, ventral, and lateral face; af - all faces together.

Sensilla		A1	A2	A3	A4	A5	A6	A7	A8	A9	Total
Chaetica C1	df	15	4	4	2	5	5	4	7	3	49
	vf	12	3	3	4	3	3	2	5	2	37
	lf	9	7	3	4	6	8	2	1	6	46
	**af**	**36**	**14**	**10**	**10**	**14**	**16**	**8**	**13**	**11**	**132**
Chaetica C2	df	-	-	8	11	4	11	9	5	3	51
	vf	-	-	5	9	7	8	4	4	2	39
	lf	-	-	7	12	6	9	7	13	5	59
	**af**	**-**	**-**	**20**	**32**	**17**	**28**	**20**	**22**	**10**	**149**
Chaetica C3	df	-	-	-	2	1	2	3	1	2	11
Trichodea	df	-	-	-	2	41	22	10	13	7	95
	vf	-	-	-	5	32	36	22	10	5	110
	lf	-	-	5	17	45	47	31	29	34	208
	**af**	**-**	**-**	**5**	**24**	**118**	**105**	**63**	**52**	**46**	**413**
Basiconica B1	df	-	-	1	28	11	18	26	23	16	123
	vf	-	-	-	13	15	23	20	17	19	107
	lf	-	-	-	2	3	7	5	8	10	35
	**af**	**-**	**-**	**1**	**43**	**29**	**48**	**51**	**48**	**45**	**265**
Basiconica B2	df	-	-	-	2	4	4	6	3	2	21
	vf	-	-	-	4	3	3	8	5	2	25
	lf	-	-	-	-	-	-	1	2	1	4
	**af**	-	-	-	**6**	**7**	**7**	**15**	**10**	**5**	**50**
Basiconica B3	df	-	-	-	-	1	3	3	2	2	11
Styloconica	df	-	-	1	1	1	1	-	1	3	8
Total		**36**	**14**	**37**	**118**	**188**	**210**	**163**	**149**	**124**	**1039**

Table S10. Numbers of sensilla on antennae of *Spermozoros weiweii*, female apteron. A1–A9 = antennomeres 1–9; df, vf, lf = dorsal, ventral, and lateral face; af - all faces together.

Sensilla		A1	A2	A3	A4	A5	A6	A7	A8	A9	Total
Chaetica C1	df	16	3	4	3	4	3	6	2	7	45
	vf	6	3	6	5	5	3	3	4	4	39
	lf	11	4	5	4	4	4	2	7	3	44
	**af**	**30**	**10**	**15**	**12**	**13**	**10**	**11**	**13**	**14**	**128**
Chaetica C2	df	-	-	5	8	3	9	11	9	6	51
	vf	-	-	3	10	7	13	6	10	3	52
	lf	-	-	9	11	5	3	10	9	4	51
	**af**	**-**	**-**	**17**	**29**	**15**	**15**	**27**	**28**	**13**	**154**
Chaetica C3	df	**-**	**-**	1	1	1	1	1	1	1	**7**
Trichodea	df	-	-	2	7	29	19	6	8	9	76
	vf	-	-	3	10	18	14	8	6	2	62
	lf	-	-	6	13	32	31	17	13	30	142
	**af**	**-**	**-**	**11**	**30**	**79**	**60**	**31**	**27**	**42**	**280**
Basiconica B1	df	-	-	3	8	7	13	35	52	35	153
	vf	-	-	1	11	11	18	39	28	7	115
	lf	-	-	4	3	8	15	10	5	2	47
	**af**	**-**	**-**	**8**	**22**	**26**	**46**	**84**	**85**	**44**	**315**
Basiconica B2	df	-	-	-	2	3	1	2	4	1	13
	vf	-	-	-	1	2	2	2	3	3	13
	lf	-	-	1	-	-	-	-	1	-	2
	**af**	-	-	**1**	**3**	**5**	**3**	**4**	**8**	**4**	**28**
Basiconica B3	df	**-**	**-**	**-**	**-**	**-**	3	2	2	2	9
Styloconica	df	-	-	1	1	-	1	1	-	1	5
Total		**30**	**10**	**54**	**98**	**139**	**149**	**161**	**164**	**121**	**926**

Table S11. Antennal sensillum (sub)types in Zoraptera and Dermaptera. Information on antennae of *Usazoros hubbardi* was taken from Slifer & Sekhon (1978), and that of *Forficula auricularia* (Dermaptera) from Faucheux (2022).

	Spiralizoros	*Spermozoros*	*Usazoros hubbardi*	*Forficula auricularia*
S. chaetica C1	+	+	B, thick-walled chemoreceptors	chaetica C2, C3
S. chaetica C2	+	+	A, tactile hairs	chaetica C1
S. chaetica C3	-	+	-	chaetica C4
S. trichodea	+	+	E, long, slender thin-walled chemoreceptors	-
S. basiconica B1	+	+	D, short, slender thin-walled chemoreceptors	-
S. basiconica B2	+	+	C, wide thin-walled chemoreceptors	basiconica
S. basiconica B3	-	+	-	basiconica
S. styloconica St	-	+	-	-
S. coeloconica	-	-	-	+

References

Faucheux, M. J. Les sensilles antennaires du Perce-oreille *Forficula auricularia* Linnaeus, 1758 (Dermaptera: Forficulidae). *Bull. Soc. Sci. Nat. Ouest Fr. (N.S.)* **44**, 36–54 (2022).

Kaláb, O., Hoffmannova, J., Packova, G., Kočárková, I., Kundrata, R. & Kočárek, P. Curated global occurrence dataset of the insect order Zoraptera. *Scientific Data* (in press).

Slifer, E. H. & Sekhon, S. S. Structures on the antennal flagellum of *Zorotypus hubbardi* (Insecta, Zoraptera). *Not. Nat.* **453**, 1–8 (1978).
